# Exploring experiences and perceptions of nursing students regarding missed nursing care in Ethiopia: a descriptive qualitative study

**DOI:** 10.1186/s12912-025-04222-2

**Published:** 2025-12-12

**Authors:** Muktar Abawaji, Rachel Cardwell, Gugsa Germossa, Lisa McKenna

**Affiliations:** 1 School of Nursing and Midwifery, Wollega University, Nekemte, Ethiopia; 2 School of Nursing and Midwifery, La Trobe University, Melbourne, Australia; 3 School of Nursing, Faculty of Health Science, Jimma University, Jimma, Ethiopia

**Keywords:** Clinical practice, Ethiopia, Missed nursing care, Nursing care omission, Nursing students, Unfinished nursing care

## Abstract

**Background:**

Missed nursing care is a critical issue, particularly in resource-limited settings. Nursing students may encounter missed nursing care during clinical placements. However, despite growing international attention, no study has explored missed nursing care from nursing students’ perspectives in Ethiopia. Therefore, this study aimed to explore Ethiopian nursing students’ experiences and perceptions of missed nursing care during clinical placements.

**Methods:**

A descriptive qualitative design was used at two universities in western Ethiopia between April and June 2024. Participants were nursing students who had completed at least one clinical placement. Considering participant diversity, data collection began with an initial estimated sample of 15 students and continued until information power was achieved, resulting in 23 participants. Individual face-to-face semi-structured interviews were conducted, and data were analysed inductively using Braun and Clarke’s six-phase thematic analysis. Ethical approval and written informed consents were obtained before interviews.

**Findings:**

Four themes emerged from the transcribed data: Nature of Missed Nursing Care, Students’ Responses to Missed Nursing Care, Consequences of Missed Nursing Care, and Overcoming Missed Nursing Care. Students frequently observed omissions in basic and complex nursing care, including hygiene, counselling, bed making, wound care, vital signs monitoring, timely medication administration, and documentation. These omissions were attributed to systemic factors such as nurse shortages, high patient load, and lack of equipment, as well as personal factors including nurses’ carelessness and negligence. Most students viewed missed nursing care as undesirable and experienced emotional distress when witnessing it, though a few perceived some omissions as minor. Students believed missed nursing care compromised patient outcomes and hindered their learning and professional development. They also suggested solutions such as improving staffing, resources, teamwork, and supervision.

**Conclusion:**

Missed nursing care is a multifaceted issue frequently encountered by nursing students during clinical placements. Addressing this problem is essential not only to enhance patient safety but also to improve students’ clinical learning experiences and better prepare them for future practice. This study provides a foundation for future research exploring how exposure to missed nursing care influences students’ professional development.

**Supplementary Information:**

The online version contains supplementary material available at 10.1186/s12912-025-04222-2.

## Introduction

Clinical practice is a crucial component of nursing education, and nursing students spend about half of their education time in clinical settings [1]. Clinical practice allow students to apply theoretical knowledge learned in classrooms to real-world clinical practice [2, 3], helping them acquire essential clinical skills and develop competence for future professional practice [4].

The nature of students’ clinical learning environments play a central role in shaping both their educational outcomes and their future approach to patient care [5]. Research indicates that clinical placements are often challenging for nursing students, and these difficulties may hinder their learning [6, 7]. Nursing students often report experiencing frustration due to discrepancies between their theoretical preparation and actual clinical practice [8], and encounter ethical dilemmas, such as whether to speak up about neglected patient care or remain silent to avoid conflict [9].

Missed nursing care has emerged as a significant challenge for nursing students. It is defined as any aspect of required nursing care that is omitted or delayed and is recognised as a widespread issue across healthcare settings and cultures [10]. Studies from various contexts illustrate the extent of this problem. For example, in Slovakia, all nursing students in one study reported witnessing at least one missed nursing task during their clinical placement [11], and another Slovak study confirmed similar recurring omissions [12]. Italian nursing students likewise observed that essential nursing tasks were frequently overlooked [13]. A recent scoping review further highlighted the growing nature of this problem among nursing students, with implications for patient well-being, student learning and professional development [14].

The international literature consistently shows that nursing students observe omissions of essential nursing tasks during clinical practice. In the United Kingdom, for instance, students reported that tasks such as patient teaching, ambulation, discharge planning, surveillance, and monitoring intake and output were frequently overlooked [15]. German students similarly noted that hygiene, comfort, mouth and skin care, patient education, emotional support, and medication administration were often missed [16]. Students attributed these omissions to structural and organisational challenges, including staff shortages, heavy workloads, ineffective leadership, shifting priorities, poor communication, and weak teamwork [15]. Consistent with these findings, Kohanová et al. (2024) reported that inadequate numbers of nurses and a high number of patients per shift contributed to care omissions [11].

Witnessing missed nursing care elicits a range of emotional and ethical responses among nursing students. Students experienced uncertainty and confusion when patients’ care needs are neglected [17], perceiving such omissions as negligence of patients’ rights and disregard for professional ethics and standards [16]. Despite these concerns, evidence from the United Kingdom indicates that some students passively accepted missed nursing care and gradually adapted to it, regardless of awareness of its negative consequences [15]. Even so, students reported that missed nursing care affected their feelings about nursing and nursing care [15].

The consequences of missed nursing care extend beyond immediate care omissions, affecting both patient outcomes and students’ professional development. Evidence from Slovakia shows that omissions in care can lead to deterioration in patients’ health and contribute to the occurrence of adverse outcomes such as falls, bed sores, and infections [8]. For students, exposure to missed nursing care has been linked to reduced learning opportunities and may hinder their ability to meet required practice standards [18]. In addition, witnessing missed nursing care led students to intrapersonal conflicts, self-doubts, feelings of guilt, fear and helplessness [16]. Furthermore, the normalisation of missed nursing care during clinical placements, as reported in the United Kingdom, potentially could lead to perpetuation of the problem by future generations of nurses [15].

In Ethiopia, systemic healthcare challenges such as high nurse-to-patient ratios, heavy workloads, and limited resources have been reported to compromise the quality of nursing care [19]. These conditions contributed to high levels of missed nursing care among registered nurses, with direct implications for patient safety [20, 21]. However, little is known about how nursing students understand and experience this phenomenon within such a constrained healthcare environment. Gaining insight into their experiences is essential, as today’s students are the future nursing workforce, and their exposure to missed nursing care may shape their learning, professional values, and future practice. Moreover, global evidence on missed nursing care among nursing students remains limited, particularly in low-income settings. This study therefore explored nursing students’ experiences and perceptions of missed nursing care during clinical placements in Ethiopia, with the aim of informing educational and clinical strategies to support student learning and improve patient outcomes.

## Methods

### Study design

This qualitative study was part of a larger concurrent mixed-methods project examining missed nursing care among nursing students. Following an initial cross-sectional survey (blinded for review), a descriptive qualitative design was used to explore nursing students’ perceptions and experiences of missed nursing care during clinical placements.

### Study settings

The study was conducted at two universities in western Ethiopia between April and June 2024. The first university was a public institution, while the second was private. The first university primarily uses its own hospital for students’ clinical placements, whereas the second typically places students in regional hospitals; both were located in Nekemte town, about 320 km from the capital city, Addis Ababa. These universities were purposively selected as they are major providers of nursing education in the region, with large student enrolments and diverse nursing programs. They were accessible to the research team, facilitating smooth ethical clearance, participant recruitment, and data collection.

### Population

The participants for this qualitative study were invited from nursing students who had completed the initial cross-sectional survey. At the end of the survey, students were asked if they would be willing to share their experiences with missed nursing care in a follow-up interview. Those who indicated their willingness to discuss their experiences with missed nursing care formed the participant pool for the study.

### Eligibility criteria

Students were eligible for inclusion if they were enrolled at one of the two universities and had completed at least one clinical placement. Additionally, students were required to have completed the initial cross-sectional survey and indicated willingness to share their experiences or observations of missed nursing care in a follow-up interview.

### Sample size determination

From students who indicated their willingness to share their experiences, an initial sample of 15 students was planned to ensure inclusion of participants with diverse characteristics across gender (male and female), program type (generic and post-basic), institution (public and private), and year of study (second to fourth year). The final sample size was determined based on the concept of information power [22, 23], which suggests that the more relevant and information-rich the data are in relation to the study aim, the fewer participants are required. As data collection progressed, interviews and preliminary analysis were conducted concurrently, allowing the researcher to assess data adequacy. Additional participants were recruited until the information obtained was sufficient to address the research objectives comprehensively. At this stage, no new insights or themes emerged, indicating that both information power and thematic saturation had been achieved. This approach ensured that the final sample was sufficient to capture the depth of students’ perceptions and experiences relevant to the study aim. The final sample comprised 23 participants, which was considered adequate to provide both depth and diversity of perspectives, and hence, information power.

### Sampling techniques

A purposive sampling strategy with maximum variation was employed to recruit students who could provide rich and relevant insights into missed nursing care. Participants were intentionally selected from the survey respondents who agreed to be contacted, ensuring diversity in academic and demographic characteristics. This approach enabled the collection of rich, relevant insights into missed nursing care and a wide range of perspectives.

### Data collection tools

A semi-structured interview guide was used to foster a participant-centred conversation. It also offered flexibility to ask follow-up questions and delve deeper into participants’ responses [24]. Questions included: Could you share your experience of missed nursing care during your last clinical placement?; How do you see omissions of nursing care?; What do you think the impact of missed nursing care is on patients?; How do you perceive the impact of missed nursing care on your overall education and professional development?, and From your experiences or observations, how do you think missed nursing care can be prevented? (Supplementary File 1)

### Data collection procedures

Students who expressed interest in sharing their experiences of missed nursing care and provided contact details were followed up. The purpose of the interview was explained, and written informed consent was obtained from each participant. Individual face-to-face interviews were then conducted and audio-recorded using a digital voice recorder [25]. Field notes were also taken to capture non-verbal cues and contextual details [26, 27]. Participants were informed that all information would be kept confidential and accessible only to authorised individuals involved in the research project. To ensure confidentiality, each participant was represented by a unique code, and all data were stored and handled securely to prevent unauthorised access. Participants were further assured that questionnaires were anonymous and contained no identifying information. Interviews were conducted in a private, quiet room on the university campuses to ensure comfort and privacy.

### Data analysis

All interview recordings were transcribed verbatim. Inductive thematic analysis was conducted following the six phases of Braun and Clarke: familiarisation with data, generating initial codes, combining codes into themes, reviewing themes, defining and naming themes, and reporting results [28]. The first author read and reread all transcripts to gain familiarity with the data and generated initial codes line by line. Codes were then grouped based on similarities to form preliminary categories, which were further refined into themes and subthemes. The themes were reviewed and discussed among the research team to ensure consistency and accurate representation of participants’ views. Final themes were defined, named, and supported by illustrative quotations from the transcripts. The analysis was conducted in the source language, and relevant quotes were translated into English by one research team member, and another team member checked and confirmed accuracy of the translation [29].

### Trustworthiness

Trustworthiness of the findings was ensured by following Lincoln and Guba’s (1985) criteria of credibility, dependability, confirmability, and transferability [30]. Credibility was achieved through close engagement with participants during the interviews, allowing the researcher to obtain rich and authentic accounts. Direct participant quotations were used to authentically represent their experiences, and peer debriefing with the supervisory team helped validate data interpretation. Transferability was supported by providing detailed descriptions of the study context, participant characteristics, and research process to allow readers to assess applicability to other contexts. Maximum variation sampling was also employed to include nursing students with diverse backgrounds, ensuring a wide range of perspectives. Dependability was maintained through the use of a consistent interview guide across all interviews conducted by the same researcher, along with an audit trail of transcripts, coding decisions, and analytic notes. Confirmability was achieved through reflexive practices and validation of translated quotations by a second author fluent in the local language, ensuring that the findings accurately reflected participants’ perspectives.

## Findings

### Participant characteristics

A total of 23 nursing students were interviewed. Their ages ranged from 21 to 38 years, with a mean of 26.3 years. Most participants were male (*n* = 17), and six were female. The post-basic program accounted for over half (*n* = 12), whereas 11 were enrolled in the generic nursing program. Table 1 presents the demographic characteristics of the study participants.

**Table 1:** Participants’ characteristics (*n* = 23)

Variables	Categories	*n* (%)
University/Institution	University 1	20 (87.0)
	University 2	3 (13.0)
Gender	Male	17 (73.9)
	Female	6 (26.1)
Marital status	Single	13 (56.5)
	Married	10 (43.5)
Program of study	Post-basic bachelor	12 (52.2)
	Generic bachelor	11 (47.8)
Academic year	Second year	16 (69.6)
	Third year	4 (17.4)
	Fourth year	3 (13.0)
Previous University/College experience	Yes	13 (56.5)
	No	10 (43.5)
Previous experience of working in healthcare	Yes	12 (52.2)
	No	11 (47.8)
Age of participants	Range: 21–38 years, Mean: 26.3 years	

### Thematic analysis results

Four main themes emerged from the data: (1) Nature of Missed Nursing Care, (2) Students’ Responses to Missed Nursing Care, (3) Consequences of Missed Nursing Care, and (4) Overcoming Missed Nursing Care. Together, these themes capture nursing students’ experiences and perceptions of missed nursing care, highlighting what was missed and why, how students responded to it, the resulting consequences, and strategies they identified for prevention. Table 2 summarises the main themes, sub-themes, and illustrative quotations.

**Table 2:** Main themes, sub-themes, and selected participant quotes

Main Theme	Sub-theme	Participant quotes
Nature of Missed Nursing Care	Disconnect between theory and practice	*“In theory we learn many nursing skills*,* but in the hospital*,* there is no sterility or proper cleaning*,* so we follow their way instead of what science says.” (P7)*
	Types of missed nursing care	*“Bed making is not done*,* counselling is not done*,* and patient hygiene is not done.” (P1)*
	Factors contributing to missed nursing care	*“Because of the high number of patients or low number of nurses*,* some care is neglected.” (P17)*
Students’ Responses to Missed Nursing Care	Moral and ethical views on missed care	*“I see omissions of care as violations of patient rights. It also affects patient respect. I feel their rights are violated. The reason is that the patient comes to receive care*,* to get appropriate care in the proper way. Similarly*,* workers [nurses] are supposed to provide the required care.” (P 21)*
	Emotional responses to missed care	*“I feel very angry when I see care being omitted. I feel discomfort because we are here to support our patients. If we do not provide required care*,* why are we here?” (P1)*
Consequences of Missed Nursing Care	Impact on patient outcomes	*“Due to the omission of care*,* patients may be exposed to other illnesses. For example*,* if medications such as antibiotics are not administered on time*,* patients may develop nosocomial infections. It can even lead to death.” (P22)*
	Impact on students’ professional development	*“When the required care is omitted*,* we lose the chance to learn what we need to. If a procedure is not performed during practice*,* we may be unable to perform it in the future.” (P10)*
Overcoming Missed Nursing Care	Resources and staffing	*“If we address the inadequacy of nurses and equipment*,* missed care can be prevented. For example*,* if there are many patients and a few nurses*,* it becomes disproportionate*,* and care gets skipped. Next*,* if there is no equipment*,* how can you work? Therefore*,* if these two things are resolved*,* I think missed care can be prevented.” (P 1)*
	Supervision and accountability	*“There should be proper monitoring. Legal-based measures must be taken against those who miss care. They should be held accountable*,* because without accountability*,* people may neglect care as they like.” (P16)*
	Teamwork and Communication	*“Teamwork is required*,* and nurses should communicate to share information about what care was provided and what is planned.” (P20)*
	Skills Development	*“I think the main reason for missed care is a skill gap. This can be reduced by providing regular training for nurses and keeping them updated.” (P22)*

#### Theme 1: Nature of missed nursing care

This theme describes the theory-practice gap, the specific nursing activities that are omitted, and the systemic and individual factors contributing to these omissions.

##### Sub-theme 1.1: Disconnect between theory and practice

Students consistently described a clear mismatch between classroom teaching and clinical reality, noting that fundamental standards they were taught were not practised in hospital settings. As one second-year male student noted:

*Here [in theory]*,* we learned a lot of nursing care*,* but nothing is there [in the hospital]. It is possible to say there is no sterility or cleaning there. So*,* when we go there*,* we follow the hospital’s way*,* not what is in science.* (P 7)

Similarly, a second-year female student reflected that even basic skills were not enacted:

*The nursing care we learned in theory and what we see when we go to hospital is very different. For example*,* we go after learning bed making*,* thinking we have to do bed making*,* but that is not there.* (P 8)

These illustrate how the theory-practice gap, not only exposed students to environments where essential care was routinely omitted, but also blurred their understandings of what appropriate nursing care should involve.

##### Sub-theme 1.2: Types of missed nursing care

Students identified a wide range of routine and essential nursing activities that were regularly omitted in clinical settings, spanning basic care, monitoring, medication administration, and patient education. These omissions affected both fundamental and more complex aspects of nursing care. As one third-year female student described:

*Bed making is not done*,* counselling is not done*,* and patient hygiene is not done. This leads us to believe that these nursing care tasks are to be provided by patient relatives.* (P 1)

Other students highlighted the omissions of clinically significant tasks, such as wound care and timely monitoring. A fourth-year male student noted:

*A patient who had experienced a car accident came in. His wound was sutured*,* and wound care was prescribed TID [three times daily]. But they provided wound care only once per day. It was prescribed three times*,* and they gave it only once. As a result*,* his wound developed an infection and an abscess.* (P 11)

Similarly, vital signs monitoring and documentation were often skipped. One second-year male student explained:

*Taking vital signs regularly is overlooked. Similarly*,* recording what patients received is usually neglected. Similarly*,* prescribed medication that should be provided on time is given after the time has elapsed.* (P 20)

Patient education, particularly in chronic illness care was also frequently neglected. A second-year male student reported:

*For patients with heart failure for example*,* there is positioning*,* restrictions on diet. No one cares about these. It is posted ‘salt free diet’ but no one checks the types of food the patient is eating. When we ask why these occur*,* they [nurses] say ‘You came to learn*,* not to teach us.’* (P 16).

These narratives indicate that missed nursing care comprised a broad range of core responsibilities, reflecting systemic issues rather than isolated lapses in practice.

##### Sub-theme 1.3: Factors contributing to missed nursing care

Students attributed omissions in care to a combination of systemic constraints and individual nurse behaviours. Structural issues, particularly nurse shortages, high patient loads, and inadequate supplies, were the most common system-level issues. As one second-year male student explained:

*Because of the high numbers of patients or low numbers of nurses*,* there cares that is neglected. For example*,* for patients admitted to hospital*,* while we were waiting for morning medications to be administered*,* there were times they are not given. This happens due to high patient load or a shortage of nurses.* (P 17)

Lack of equipment or supplies also hindered the delivery of nursing care. A second-year male student described:

*Last time*,* when we were in a medical ward*,* there was no NG [nasogastric] tube for insertion. The patient needed an NG tube*,* but it was not available. This is not the nurses’ fault or the patient’s fault; it is a problem with the hospital’s supply.* (P 7)

In addition to systemic pressures, some participants highlighted nurse-related factors, including negligence and dismissive attitudes. As one second-year male student noted:

*Carelessness is seen on the nurses’ side. Even when patients ask for care that should be provided*,* there are times they are yelled at and demeaned.* (P 5)

The overall stories reveal that missed nursing care resulted from both structural deficits and individual nurse behaviours, illustrating the multifactorial nature of the problem.

#### Theme 2: Students’ responses to missed nursing care

This theme focuses on how nursing students perceived missed nursing care, both in terms of their ethical judgements and their emotional reactions.

##### Sub-theme 2.1: Moral and ethical views on missed nursing care

Students commonly regarded missed nursing care as ethically unacceptable and a violation of patients’ rights. They expressed a strong belief that nurses have a moral duty to provide complete and timely care. As one second-year male student stated:

*A person [patient] does not come to us [hospital] for recreation; they come sick. This person needs care from us*,* next to God. Providing that care is essential. However*,* some individuals [nurses] miss a lot of care as a result of their selfishness. For me*,* their missing care is a big mistake; it has a severe impact on patients and on the nurses themselves. (P 20)*

Other participants framed omissions of nursing care as breaches of patient rights and dignity. For example, a third-year male student explained:

*I see omissions of care as violations of patient rights. It also affects patient respect. I feel their rights are violated. The reason is that the patient comes to receive care*,* to get appropriate care in the proper way. Similarly*,* workers [nurses] are supposed to provide the required care for that patient. (P 21)*

Similarly, a fourth-year female student expressed her belief:

*Complete nursing care must be provided for admitted patients. If required nursing care is omitted*,* the patient may face other complications*,* it has serious consequences. Therefore*,* I believe that complete nursing care must be provided to a patient; missing care is wrong. (P 3)*

A few students, however, acknowledged that not all omissions cause serious harm. One fourth-year female student noted:

*Missing care is not appropriate*,* but there are conditions where it has no problem. There are also conditions where it may even lead to death. So*,* those that should not be missed should not be missed*,* and the main ones should be provided. […] Missing some care may not lead to a problem. (P 6)*

Taken together, these accounts reflect that missed nursing care was not simply a technical lapse, but an ethical failure that undermined patient rights and professional integrity.

##### Sub-theme 2.2: Emotional responses to missed nursing care

Witnessing missed care evoked strong emotional reactions among students, including anger, sadness, frustrations, and, for some, a sense of hopelessness. These emotions shaped their identity formation and commitment to the profession. A third-year female student described intense frustration:

*I feel very angry when I see nursing care being omitted. The reason is that when the patients do not get what they expected from us and came for*,* I do not feel peace internally. There is a lot of discomfort because we are here to support our nation. […] We are nurses—if we do not do this [provide required care]*,* why are we here? (P 1)*

Others felt deep sadness and empathy for patients. A fourth-year female student said:

*I feel very sad when a person does not receive the nursing care they should get. I also feel deeply sad thinking about the patient and imagining what if it were my family or me. (P 3)*

For some students, repeated exposure to missed care led them to question their career choice. For example, as one fourth-year male student recalled:

*There was a time when I said why I chose nursing. When it was written and posted on the wall that wound care should be provided three times a day*,* and nurses did not provide this care for the patient*,* I truly felt a loss of hope. There were moments when I thought*,* ‘Why did I study? I should not have joined this department’. (P 11)*

These narratives illustrate that missed nursing care had profound emotional impacts on students, shaping their professional identities and challenging their motivation and commitment to nursing.

#### Theme 3: Consequences of missed nursing care

Students described wide-ranging consequences of missed nursing care, highlighting its detrimental effects on patient outcomes as well as the professional development of nursing students.

##### Sub-theme 3.1: Impacts on patient outcomes

Students highlighted that missed nursing care directly affected patients’ health, recovery, and safety. For example, a third-year male student explained:

*Due to the omission of necessary care*,* patients may be exposed to other illnesses. For example*,* if medications such as antibiotics are not administered on time*,* patients may develop nosocomial infections. It can also lead patients to death. (P 22)*

Similarly, a second-year male student recalled:

*I remember a patient with CHF [Congestive Heart Failure] who was admitted to the hospital and stayed there for about three months. She developed fourth-degree bedsores because nurses failed to reposition her. (P 7)*

Students also associated missed nursing care with delayed recovery, prolonged hospital stays, and financial strain on patients. As one second-year male student stated:

*Missed nursing care can prevent the patient from recovering quickly. When a patient stays in the hospital for a long time*,* they may be exposed to nosocomial infections. While in the hospital*,* the patient pays for the bed*,* materials*,* and medication*,* and these expenses can have an economic impact on them. (P 2)*

These accounts illustrate that missed nursing care contributed to preventable complications, prolonged recovery, and increased financial burden, reflecting its broader implications for patient safety and quality of care.

##### Sub-theme 3.2: Impacts on students’ professional development

Students explained that missed nursing care limited their opportunities to observe and practice essential nursing procedures, hindering their skill development. For instance, one third-year male student mentioned:

*If the required care is omitted*,* we miss the opportunity to learn what we need to. For example*,* if a procedure is not performed during practice*,* we do not have the chance to observe it*,* and we may be unable to perform it in the future. If you do not observe and practice during practicum*,* you will not be able to perform it in the future. (P 10)*

Witnessing missed nursing care also negatively influenced students’ motivation and attitudes toward the profession. A male second-year student expressed:

*We go to hospitals to observe in practice what we have learned in theory. When what we learned in theory is not reflected in practice*,* we lose hope*,* and our attitude toward the profession decline. (P 5)*

Some students expressed concern that repeated exposure to missed nursing care could normalise such behaviour and influence future nursing practice. As a second-year male student explained:

*People resemble where they have been. This means when we witness care being missed and live in that environment*,* we will do the same when we start working. This is because we grew up observing it. Children grow up observing their families*,* right? Therefore*,* we will perform tomorrow what we see and observe today. (P 7)*

Overall, the narratives highlight that missed nursing care undermined students’ learning opportunities, weakened their professional identity formation, and risked perpetuating substandard practices into their future careers.

#### Theme 4: Overcoming missed nursing care

This theme captures students’ reflections on strategies to prevent missed nursing care. Their suggestions centred on improving resources and staffing, strengthening supervision and accountability, fostering teamwork and communication, and enhancing opportunities for skill development.

##### Sub-theme 4.1: Resources and staffing

Students emphasised that adequate staffing levels and sufficient equipment are critical to preventing omissions in care. Many students noted that nurses’ workloads and limited material resources made it difficult to provide timely and complete care. As a second-year male student explained:

*The adequacy of numbers of professionals [nurses] makes care not missed. A low number of professionals [nurses] has a great impact on the delivery of care. Therefore*,* there should be an adequate number of professionals [nurses]. (P 17)*

A third-year female student added:

*If we address the inadequacy of nurses and equipment*,* missed care can be prevented. For example*,* if there are many patients and a few nurses*,* it becomes disproportionate*,* and care gets skipped. Next*,* if there is no equipment*,* how can you work? Therefore*,* if these two things are resolved*,* I think missed care can be prevented. (P 1)*

These narratives indicate that missed care was primarily a structural issue, suggesting that adequate staffing and material resources are essential for safe and complete delivery of care.

##### Sub-theme 4.2: Supervision and accountability

Students highlighted that closer oversight and a clear accountability system are crucial to reducing care omissions. They believed that stronger managerial involvement would help ensure that required care is consistently delivered. A male second-year student stated:

*There should be monitoring of nurses by the manager. The manager should strengthen this monitoring. I believe there should be monitoring of what patients coming to the hospital are receiving and how nurses are delivering the required care from them. (P 2)*

Similarly, a second-year male student explained:

*There should be appropriate monitoring and control. Appropriate and legal-based measures must be taken on those who miss care. They should be held accountable*,* and there should be accountability. If there is no accountability*,* people may neglect as they like. (P 16)*

These reflections suggest that supervision and accountability mechanisms were critical to promote professional responsibility and reduce the likelihood of neglecting essential care.

##### Sub-theme 4.3: Teamwork and communication

Students identified teamwork and effective communication, both among nurses and with physicians, as central to preventing care omissions. They emphasised the need for collaborative practice, information sharing, and coordinated handover process. For instance, one second-year male student explained:

*When a person [patient] is sick and comes to a healthcare facility*,* it is not one person [nurse] who provides care. Therefore*,* teamwork is required. Nurses should work as a team*,* and teamwork should be strengthened. Nurses need to meet and communicate. When a patient is transferred from one ward to another*,* they [nurses] should communicate to share information*,* ‘This care was provided to the patient yesterday*,* this was given today*,* and this is needed and planned for the future.’ (P 20)*.

Similarly, a fourth-year male student expressed:

*There are times when nurses wait for physicians’ orders*,* and times when physicians expect nurses to act autonomously. In between*,* the nursing care that patients should receive is missed. Communication between nurses and physicians*,* as well as among nurses*,* should be strengthened. (P 11)*

These reflect that missed nursing care can be prevented by stronger collaboration and more reliable communication systems that support continuity of care.

##### Sub-theme 4.4: Skills development

Students also emphasised the importance of ongoing professional development to address skill gaps that contribute to missed care. They believed that continuous training and upskilling would enhance nurses’ competence in delivering complete care. For example, a third-year male student explained:

*The first reason for missed care is a skill gap or lack of skill. So*,* if we work on the nurses’ skill gap*,* we can solve it to some extent. It [skill gap] can be reduced by providing regular training for nurses and keeping them updated. (P 22)*

These insights reveal that skill enhancement and continuous professional development were key strategies to strengthen clinical capacity and reduce omissions in care.

## Discussion

This study explored Ethiopian nursing students’ experiences and perceptions of missed nursing care during their clinical practice. The findings revealed that students perceived noticeable gaps between theoretical instruction and clinical practice, resulting in the omission of several nursing care activities. This indicates inconsistencies between what is taught in classrooms and what is practised in healthcare settings, which may blur students’ understandings of what appropriate nursing care should involve and may hinder their abilities to integrate theoretical knowledge into practice. These findings highlight the importance of strengthening linkages between classroom teaching and clinical practice to help students apply theoretical knowledge effectively. Similar findings have been reported in Slovakia and Iran, where students noted discrepancies between theoretical learning and clinical practice [8, 17]. This study adds insights by providing evidence of theory-practice gaps in a low-resource context, highlighting challenges not extensively documented in existing literature.

Students identified frequent omissions of both basic and complex nursing tasks, including wound care, personal hygiene, patient education, and timely medication administration. This finding indicates that essential aspects of patient-centred care are not being consistently delivered, reflecting systemic issues rather than isolated lapses in practice. Such omissions are particularly concerning because they can compromise patient safety [31]. The finding highlights the need for strategies that ensure all aspects of nursing care are provided. Previous studies from Australia and Slovakia have also highlighted personal hygiene as one of the most frequently omitted care activities, while research in Germany identified wound dressing, skin care, and medication administration as commonly missed nursing tasks [8, 16, 18]. The detailed documentation of both basic and complex care omissions in the current study extends current knowledge by highlighting context-specific patterns of care omissions.

In this study, students attributed missed nursing care to both systemic and individual nurse-related factors. System-level issues such as nurse shortages, patient overload, and time constraints were described as major barriers to the consistent delivery of care. This suggests that inadequate staffing and high patient-nurse ratios may overwhelm nurses, forcing them to prioritise urgent tasks over routine but essential care. Addressing workforce shortages and reducing workload are therefore critical to improving care quality and ensuring that essential nursing tasks are not overlooked. In addition to systemic challenges, students also identified individual nurse-related factors such as nurses’ carelessness and negative attitudes toward their work as contributing to missed nursing care. This illustrates the interplay between organisational constraints and professional accountability. Comparable findings were reported in studies from the United Kingdom and Slovakia, where staff shortages, time constraints, and heavy workloads were contributors to missed nursing care [11, 15]. However, the identification of nurse-related behavioural factors in the present study appears to be a unique finding, warranting further investigation. These findings highlight dimensions of missed nursing care in low-resource contexts, where both organisational and individual factors intersect, indicating a multifactorial nature of the problem.

Students in this study believed that all required nursing care should be delivered and perceived missed nursing care as a violation of patient rights that could have legal consequences. This finding reflects a strong sense of professional responsibility, with students recognising that omissions in care undermine patient dignity and violate professional standards. This indicates that omission of care is an ethical failure that undermines patient rights and professional integrity. A previous study from Germany similarly reported that students viewed missed nursing care as compromising patient dignity and professional standards [16]. However, some students in the current study perceived that certain missed nursing care tasks might not cause immediate harm to patients, reflecting that they may begin to rationalise and normalise missed nursing care. This finding indicates a need to address the mindset of rationalising and normalising of care omissions. A similar perspective was reported in the United Kingdom, where students noted that missed nursing care did not kill patients and did not result in short-term negative consequences [15].

Students reported experiencing a range of emotional reactions when witnessing missed nursing care, including anger, sadness, disappointment, frustration, hopelessness, and regret regarding their choice of the nursing profession. These experiences suggest that exposure to missed nursing care can negatively affect students’ attitudes toward nursing, potentially impacting motivation, commitment, and professional identity. Providing students with guidance, mentorship, and emotional support during clinical practice may help mitigate these negative effects and maintain professional engagement. There is also a need for educational and institutional support to help students cope with the challenges of clinical practice. Similar experiences have been reported in Germany, the United Kingdom, and Slovakia, where students described frustration, intrapersonal conflict, and distress when observing missed nursing care [8, 15, 16]. This study provides a new insight into the psychological and emotional burden of care omissions on students in a resource-limited setting.

Students reflected that omissions in nursing care compromised patient health and well-being, leading to adverse outcomes such as infections, complications, and even death. This indicates that missed nursing care can have serious impacts on patient safety and quality of care and suggests the importance of consistently performing essential nursing tasks. In addition, students reported that missed nursing care may result in delayed recovery, prolonged hospital stays, increased healthcare costs, and reduced patient satisfaction with healthcare services. A previous study from Slovakia reported similar negative impacts of missed nursing care on patient health, including pressure ulcers, falls, and infections [8]. However, some of the broader consequences noted in the current study, such as effects on hospital costs and patient satisfaction, have not been widely documented in previous literature. This expands the understanding of missed nursing care impacts beyond clinical outcomes, offering new insights into its economic and experiential consequences.

Beyond patient outcomes, students reported that missed nursing care affected their learning and professional development, potentially limiting their competence in essential nursing skills due to reduced hands-on exposure. This suggests that repeated exposure to missed nursing care may hinder students’ abilities to meet practice standards and develop clinical competencies. Additionally, some students noted that observing missed nursing care could lead them to accept it as part of the professional culture, raising concerns that such practices might be perpetuated by future generations of nurses. Similar findings have been reported in Australia and Pakistan, where students reported exposure to missed nursing care negatively affected their learning and professional socialisation [9, 18]. Studies from the United Kingdom and Italy also support the current study finding, showing that students may replicate missed nursing care observed from senior nurses, thereby continuing care omissions in future practice [15, 32]. This finding highlights the importance of targeted interventions to ensure students internalise proper nursing standards and avoid normalising care omissions. The current study’s findings contribute new insights by showing how exposure to missed nursing care undermines students’ learning opportunities, shape their professional identity formation, and risks perpetuating substandard practises.

Students proposed several strategies to reduce missed nursing care, including ensuring sufficient nurse staffing, providing proper supervision and monitoring, promoting effective teamwork and communication, and offering regular training and ongoing professional development. Implementing these strategies may enhance the quality of nursing care, improve patient outcomes, and support students’ professional growth. These suggestions indicate that students are active observers and can identify practical solutions to improve nursing care delivery. Evidence from Slovakia, the United Kingdom, and Australia supports the current study’s recommendations, showing that adequate staffing, organised management, effective teamwork, and continuous education can reduce nursing care omissions [15, 18, 33]. This finding highlights the importance of engaging nursing students not only as learners but also as contributors to improving clinical practice standards.

Overall, this study contributes to the growing body of knowledge on missed nursing care by providing qualitative evidence from a low-resource context, focusing on the perspectives of nursing students. It extends current understanding by revealing behavioural dimensions, educational implications, and systemic challenges specific to clinical learning environments in Ethiopia.

## Implications of the study

The findings of this study have important implications for nursing education, clinical training, and healthcare policy in Ethiopia. The reported theory-practice gap highlights the need to strengthen integration of classroom instruction with clinical practice. Nursing educators should enhance clinical supervision, reflective learning, and practical mentorship to help students translate theoretical knowledge into clinical practice. The identification of systemic and individual factors contributing to missed nursing care indicates the importance of addressing workforce challenges, including nurse staffing, workload management, and professional accountability. Healthcare institutions should prioritise resource allocation and foster supportive clinical environments. Policies promoting teamwork, ongoing professional development, and adherence to care standards are essential to maintain patient safety and quality of care.

The emotional and professional challenges experienced by students indicate that clinical education should include support systems that address psychological well-being and professional socialisation. Structured mentorship, debriefing sessions, and training on task prioritisation can help students manage the pressures of clinical practice and maintain professional engagement. Students’ recommendations regarding teamwork, supervision, and continuing education demonstrate that learners can actively contribute to improving clinical care. Policymakers and educational leaders should engage students as partners in designing interventions aimed at reducing missed nursing care, fostering professional responsibility, and enhancing patient safety. Collectively, these implications provide actionable strategies to strengthen nursing education, improve clinical practice, and inform policy reforms, ultimately supporting safer, higher-quality patient care in low-resource settings.

## Limitations of the study

This study has several limitations that should be considered when interpreting the findings. First, participants were asked to recall their experiences of missed nursing care during previous clinical placements, which may have introduced recall bias. Some details or experiences may have been forgotten or reconstructed over time, potentially affecting the findings. Second, social desirability bias may have influenced participants to present themselves in a more favourable light. Given the sensitive nature of missed nursing care, students might have underreported instances in which they were personally involved or placed greater emphasis on organisational factors. This could have shaped the findings toward more socially acceptable or system-focused explanations, thereby influencing interpretation. Although the study purposively included participants with diverse characteristics, voluntary participation may have led to underrepresentation of some viewpoints, such as those of students less willing to share negative experiences. Lastly, the study focused solely on nursing students, and perspectives of clinical staff were not included, as the aim was to understand students’ experiences, feelings, and the potential influence on their learning and future professional practice. While this focus allowed for an in-depth exploration of the students’ viewpoint, it limits the transferability of the findings to broader clinical contexts. Future studies could mitigate these limitations by triangulating data with other sources, such as interviews with nurses, to provide more comprehensive understandings of missed nursing care.

## Conclusions

Nursing students frequently observed omissions in both basic and complex nursing care during clinical practice, driven by systemic constraints and individual nurse-related factors. These omissions compromised patient outcomes, hindered students’ learning, and affected their professional development. Students identified strategies to reduce missed nursing care, including improved supervision, teamwork, resource allocation, and ongoing professional development. These findings highlight the need for nursing education and healthcare systems in Ethiopia to strengthen the integration of theory and practice, provide structured support during clinical placements, and address systemic barriers to care delivery. Stakeholders should implement targeted interventions to reduce missed nursing care, foster professional identity formation, and ensure safe, patient-centred nursing practice. Prioritising these actions will advance nursing education and clinical practice, supporting safer, higher quality care and the growth of future nurses. This study provides a foundation for nursing education researchers to further investigate how nursing students’ experiences of missed nursing care during clinical placements influence their clinical learning and professional development.

## Supplementary Information

Below is the link to the electronic supplementary material.

Supplementary Material 1

## Data Availability

The data that support the results of this study are available from the corresponding author upon request.
