# Supplementary material for: Exploring experiences and perceptions of nursing students regarding missed nursing care in Ethiopia: a descriptive qualitative study
Source: BMC Nurs. 2025 Dec 12;25:58. doi: 10.1186/s12912-025-04222-2 (PMC12817531; doi:10.1186/s12912-025-04222-2)
Supplement: Supplementary file 1 — Supplementary Material 1 [file 12912_2025_4222_MOESM1_ESM.docx]

**SEMI-STRUCTURED INTERVIEW GUIDE**

**Introduction**

This interview aims to explore your experiences and perceptions regarding missed nursing care during your last clinical placement. In this interview, you will be asked about how you experienced missed nursing care, your perception of its impacts and prevention strategies. We would like to ask you to engage in a conversational manner, providing detailed accounts or examples when discussing the topic. Your insights are valuable in understanding the complexities surrounding this topic.

Interviewee code: ________________, Gender: __________________

Program of study: ______________, Year of study: ________________

**Part I: Exploring personal experiences of students on missed nursing care**

1. Could you share with me your experience of missed nursing care during your last clinical placement?

**Probe**: From your experience, what types of nursing care are most often missed? What do you think are the reasons for this?

**Probe**: How did you feel when this happened? What was going through your mind at that time?

**Part II: Perceived impacts of missed nursing care on patient outcomes**

1. In your view, what impacts does missed nursing care have on patients?

**Probe**: Can you recall a situation where you observed this impact during your placement?

**Part III: Perceived impacts of missed nursing care on students’ education & professional development**

1. How do you perceive the impact of missed nursing care on your overall education and professional development as a future nurse?

**Probe**: In what ways has this affected your learning, confidence or outlook?

**Part IV: Strategies for prevention of missed nursing care**

1. From your experiences or observations, how do you think missed nursing care can be avoided or prevented?

**Probe**: Can you suggest changes in the clinical environment that would help prevent missed nursing care?

**Any Additional Comments:**

1. Is there anything else you would like to add regarding your experiences or thoughts on missed nursing care during your clinical placement?

--------------------------------------------------------------------------------------------------------------------------------------------------------------------------------------------------------------------------------------------------------------------------------------------

*We sincerely thank you for your time and valuable contribution!*
